# Identification of Lipomatous Metaplasia in a Cortisol-secreting Adrenocortical Adenoma Treated With Mifepristone

**DOI:** 10.1210/jcemcr/luae151

**Published:** 2024-10-07

**Authors:** Abhinav K Rao, Trang Minh Thi Nguyen, Jenna Brennan Magri, Joseph Wolfgang Mathews

**Affiliations:** Trident Internal Medicine, Trident Medical Center, North Charleston, SC 29406, USA; Trident Internal Medicine, Trident Medical Center, North Charleston, SC 29406, USA; Department of Pathology and Laboratory Medicine, Medical University of South Carolina, Charleston, SC 29425, USA; Palmetto Endocrinology, Palmetto Primary & Specialty Care Physicians, Summerville, SC 29483, USA

**Keywords:** lipomatous metaplasia, ACTH-independent Cushing, mifepristone, adrenal cortical adenoma

## Abstract

Adrenal adenomas are benign tumors of the adrenal cortex that may secrete excess hormones, such as cortisol. They are most commonly discovered during imaging studies for unrelated problems. Lipomatous metaplasia is a rare degenerative change in adrenal adenomas, characterized by the presence of adipose tissue and hematopoietic elements within the tumor. In this report, we present a case of an adrenal adenoma with lipomatous metaplasia in a patient with hypertension, hyperlipidemia, and type II diabetes mellitus. The discovery of this adrenal mass was prompted by an evaluation of the patient's progressive hirsutism. The tumor was found to be secreting cortisol, leading to Cushing syndrome. The patient subsequently underwent surgical resection of the mass after being treated with mifepristone. The histopathological examination confirmed it to be an adrenal cortical neoplasm with lipomatous metaplasia, characterized by uncertain malignant potential. The patient did well postoperatively. Three months after left adrenalectomy, the patient's hirsutism, A1c, and hypertension improved, allowing a reduction in antihypertensives. Her body mass index stabilized, her triglyceride decreased, and her dehydroepiandrosterone sulfate level normalized. She continued to do well at follow-up visits. Overall, this was a rare case of a functioning adrenal adenoma with lipomatous metaplasia, presenting both diagnostic and therapeutic challenges.

## Introduction

The ACTH-independent endogenous Cushing syndrome is most commonly caused by primary unilateral adrenocortical tumors, either by adenomas or carcinomas. Lipomatous tumors of the adrenal gland are a diverse group of tumors composed of adipose tissue (1). Lipomatous tumors are usually benign (2) and nonfunctioning, accounting for 5% of all primary adrenal tumors. Since metaplasia plays a crucial role in the precancerous development of tissues, discovering lipomatous metaplasia within an adrenal adenoma is noteworthy, emphasizing the need to identify potential, associated risk factors. Mifepristone is 1 of the medications indicated for the treatment of ACTH-independent endogenous Cushing syndrome, especially when surgery is delayed or contraindicated. To our knowledge, no prior surgical pathology reports exist documenting adrenal adenoma with lipomatous metaplasia in the context of mifepristone use. Therefore, we present this case report to draw attention to our novel finding, treatment regimen, and patient outcomes.

## Case Presentation

A 69-year-old woman, previously diagnosed with estrogen and progesterone receptor-positive T1cNoMo invasive ductal carcinoma, had undergone a partial mastectomy, breast reconstruction, and radiotherapy and was currently receiving aromatase inhibition therapy. She presented with a range of pre-existing conditions, including obstructive sleep apnea, type 2 diabetes, hypertension, and hyperlipidemia. In the past 5 years, she had experienced a progressive history of poorly controlled hyperglycemia, diaphoresis, anxiety, easy bruising, abdominal weight gain, and hirsutism.

## Diagnostic Assessment

**Figure 1.**
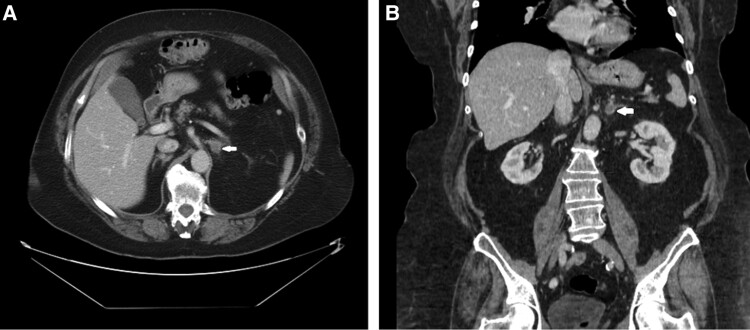
(A) CT scan axial showing a left adrenal mass as identified by the white arrow. (B) CT scan coronal view showing a left adrenal mass as identified by the white arrow.

Abbreviation: CT, computed tomography.

On clinical assessment, she was found to have central obesity (body mass index 39.6 kg/m²) and hirsutism on her face, chest, and arms. She had a blood pressure of 131/84, heart rate of 74, temperature of 36.4 °C, and oxygen saturation of 98%. Her home medications included aspirin 81 mg daily, acarbose 25 mg daily, anastrozole 1 mg daily, wellbutrin 150 mg twice a day, vitamin D3 25 mcg daily, and celebrex 200 mg daily. Additionally, her total testosterone was normal at 2.1 nmol/L (61 ng/dL; reference range: 0.5 to 2.4 nmol/L or 15 to 70 ng/dL), and her dehydroepiandrosterone sulfate (DHEA-S) was elevated at 11 240 µg/dL (reference range: 13-130 µg/dL; Table 1). Her androstenedione LCMS was normal at 3.3 nmol/L (94 ng/dL; reference range: 30-200 ng/dL), hemoglobin A1c was 7.0%, ACTH was 2.3 pmol/L (10.8 pg/mL; reference range: pmol/L 2.2-13.2 pmol/L or 10 to 60 pg/mL), and 8 Am cortisol was 14.3 µg/dL (394 nmol/L; reference range: 5-25 µg/dL or 140-690 nmol/L). Workup for hypercortisolism demonstrated a nonsuppressed cortisol with a 1 mg dexamethasone suppression test (DST) (cortisol level of 135 nmol/L, or 4.9 µg/dL; reference range: < 50 nmol/L or < 1.8 µg/dL) and a serum dexamethasone level of 10.4 nmol/L (462 µg/dL, reference range: < 50 nmol/L or < 1.8 µg/dL, Table 2). Additionally, 1 out of 3 late-night salivary cortisol levels were elevated: 0.04 µg/dL (1.1 nmol/L), 0.04 µg/dL (1.1 nmol/L), and 0.06 µg/dL (1.7 nmol/L; reference range: 0.054-0.1827µg/dL). Computed tomography abdomen and pelvis with intravenous contrast revealed a 1.1 cm left adrenal adenoma (Fig. 1).

**Table 1.** Summary of laboratory data on initial visit

Laboratory markers	Values (SI units)	Reference
HbA1c	7%	4.5-5.4%
Cholesterol	181 mg/dL (4.68 mmol/L)	0-199 mg/dL (0-5.15 mmol/L)
Triglycerides	84 mg/dL (0.95 mmol/L)	0-149 mg/dL (0-1.69 mmol/L)
HDL-C	41 mg/dL (1.06 mmol/L)	>50 mg/dL (>1.30 mmol/L)
LDL-C	123 mg/dL (3.18 mmol/L)	0-100 mg/dL (0-2.59 mmol/L)
Non HDL-C	140 mg/dL (3.62 mmol/L)	<130 mg/dL (<3.37 mmol/L)
Progesterone	0.61 ng/mL (1.94 nmol/L)	< 1 ng/mL (<3.18 nmol/L)
Sodium	138 mEq/L (138 mmol/L)	135-145 mEq/L (135-145 mmol/L)
Potassium	4.1 mEq/L (4.1 mmol/L)	3.5-5.1 mEq/L (3.5-5.1 mmol/L)
Chloride	102 mEq/L (102 mmol/L)	97-108 mEq/L (97-108 mmol/L)
Creatinine	0.70 mg/dL (62 µmol/L)	0.6-1.3 mg/dL (53-115 µmol/L)
BUN	28 mg/dL (10.0 mmol/L)	7-25 mg/dL (2.5-8.9 mmol/L)
Glucose	152 mg/dL (8.4 mmol/L)	65-99 mg/dL (3.6-5.5 mmol/L)
GFR	70 mL/min/1.73m²	>60 mL/min/1.73m²
Vitamin B12	267 pg/mL (197 pmol/L)	180-914 pg/mL (133-674 pmol/L)
Albumin/creatinine ratio in urine	2.8 µg/mg creatinine	0-29.9 µg/mg creatinine
Estradiol	19 pg/mL (70 pmol/L)	0-30 pg/mL (0-110 pmol/L)
Free testosterone (direct)	7.4 pg/mL (25.6 pmol/L)	0.0-4.2 pg/mL (0.0-14.6 pmol/L)
Total testosterone	61 ng/dL (2.1 nmol/L)	3-41 ng/dL (0.1-1.4 nmol/L)
Androstenedione LCMS	94 ng/dL (3.29 nmol/L)	17-99 ng/dL (0.59-3.46 nmol/L)
DHEA-Sulfate	308 µg/dL (8.32 mmol/L)	20.4-186.6 µg/dL (0.55-5.05 mmol/L)
FSH	19.73 mIU/mL	Postmenopausal 16.74-5.12 mIU/mL
LH	15.11 mIU/mL	Postmenopausal 10.8-58.64 mIU/mL
TSH	1.36 mIU/mL	0.45-5.33 mIU/mL
Free T4	0.77 ng/dL (9.9 pmol/L)	0.58-1.64 ng/dL (7.5-21.0 pmol/L)
Free T3	3.0 pg/mL (4.6 pmol/L)	2.5-3.9 pg/mL (3.9-6.0 pmol/L)

Abbreviations: BUN, blood urea nitrogen; DHEA-S, dehydroepiandrosterone sulfate; GFR, glomerular filtration rate; HbA1c, hemoglobin A1c; HDL-C, high-density lipoprotein cholesterol; LDL-C, low-density lipoprotein cholesterol.

**Table 2.** Laboratory data for left adrenal adenoma

A. Low-dose dexamethasone testing			
Test	Before DST	After DST	Reference range
Random a.m. cortisol	14.3 µg/dL (394.7 nmol/L)	4.9 µg/dL (135.3 nmol/L)	4.5-21 µg/dL (124.3-580.3 nmol/L)
ACTH	10.8 pg/mL (2.4 pmol/L)	7.2 pg/mL (1.6 pmol/L)	7.2-63.3 pg/mL (1.6-14.0 pmol/L)
Dexamethasone	—	462 ng/dL (11.9 nmol/L)	-

B. Adrenal cortical hormonal laboratory data		
Test	Value (SI units)	Reference range (SI units)
Late-night salivary cortisol #1	0.4 µg/dL (1.0 nmol/L)	Midnight <0.01-0.09 µg/dL (0.3-2.5 nmol/L)
Late night salivary cortisol #2	0.36 µg/dL (10.0 nmol/L)	Midnight <0.01-0.09 µg/dL (0.3-2.5 nmol/L)
Late-night salivary cortisol #3	0.06 µg/dL (1.6 nmol/L)	Midnight <0.01-0.09 µg/dL (0.3-2.5 nmol/L)
Urinary free cortisol 24 hours	13 µg/24 hours (35.9 nmol/24 hours)	6-42 µg/24 hours (16.6-116.0 nmol/24 hours)
Plasma renin activity	2.77 ng/mL/hr (0.30 ng/L/s)	0.17-5.38 ng/mL/hour (0.02-0.60 μg/L/s)
Aldosterone	29.2 ng/dL (0.8 nmol/L)	0.0-30.0 ng/dL (0.0-0.8 nmol/L)
Dopamine	<30 pg/mL (<198.0 pmol/L)	0-48 pg/mL (0-318.4 pmol/L)
Epinephrine	26 pg/mL (139.2 pmol/L)	0-62 pg/mL (0-332.2 pmol/L)
Norepinephrine	378 pg/mL (2231.4 pmol/L)	0-874 pg/mL (0-5159.8 pmol/L)
Metanephrine	27.2 pg/mL (145.6 pmol/L)	0.0-88.0 pg/mL (0.0-471.2 pmol/L)
Normetanephrine	52.8 pg/mL (310.8 pmol/L)	0.0-285.2 pg/mL (0.0-1678.8 pmol/L)
DHEA-sulfate	158 µg/dL (3.9 μmol/L)	20-187 µg/dL (0.5-4.6 μmol/L)

Abbreviations: DHEA, dehydroepiandrosterone; DST, dexamethasone suppression test.

## Treatment

Based on the patient's presentation, biochemical, and radiologic findings, she was diagnosed with a functional left adrenal tumor. Congenital adrenal hyperplasia was ruled out biochemically (previously normal 17-hydroxyprogesterone and androstenedione). She was evaluated for left adrenalectomy. However, due to delayed surgical treatment, medical therapy was started with oral mifepristone 300 mg daily and titrated up to 600 mg daily after 2 weeks. At this dosage, she demonstrated improvements in her weight and glycemic control. She was treated with oral spironolactone 100 mg daily to maintain potassium levels. She continued therapy with mifepristone and spironolactone for 4 months before holding these medications for 2 weeks before undergoing a robot-assisted laparoscopic left adrenalectomy.

## Outcome and Follow-up

**Figure 2.**
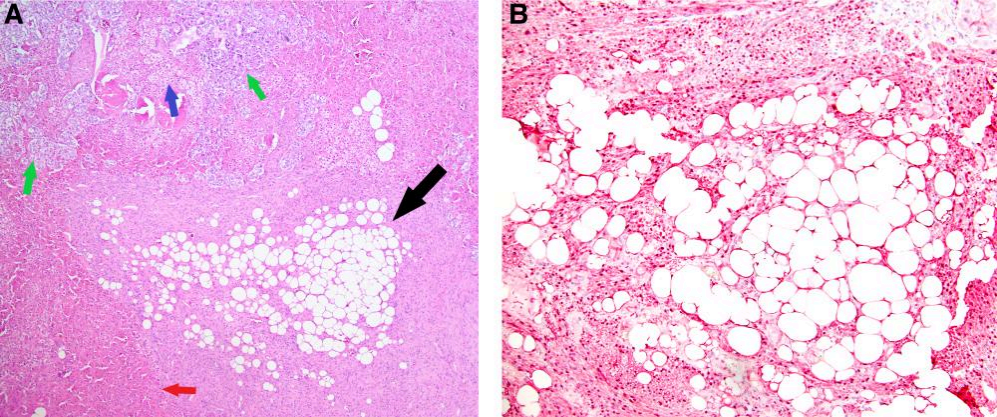
Histopathologic images. (A) Focal lipomatous metaplasia consisting of a focus of mature adipocytes (black arrow) in a background of benign-appearing adrenal cortex and medulla made of cells with clear cytoplasm and small nuclei of the zona fasciculata (blue arrow), compact eosinophilic cells of the zona reticularis (red arrow), and granular basophilic chromaffin cells of the adrenal medulla (green arrows) (hematoxylin and eosin; 40x). (B) Positive S100 immunohistochemical staining of the adipocytes making up the focal lipomatous metaplasia (100x).

Her postoperative course was uneventful. She was not treated with postoperative steroids and never demonstrated any signs of adrenal insufficiency. Grossly, the nodule appeared hemorrhagic and friable with orange-yellow irregularly shaped adrenal gland surrounded by peri-adrenal adipose tissue with no distinct areas of nodularity or hyperpigmentation. Microscopically, there was benign adrenal tissue with scattered microscopic foci of somewhat nodular hyperplastic growth. There was an ill-defined potential dominant nodule that measured approximately 1.2 cm in the greatest dimension. Additionally, there was focal lipomatous metaplasia in 1 micronodule. The histologic features of the nodular areas were assessed according to Weiss criteria. No adverse features were identified (score = 0), which favored a benign process (Fig. 2). Seven weeks after surgery, her DHEA-S and ACTH levels normalized (103 µg/dL and 59 pg/mL, respectively).

Three months after her left adrenalectomy, her hirsutism improved, her A1c improved to 6.7%, her hypertension improved with a reduction in her antihypertensive medications, and her body mass index remained at 36 kg/m2. Her DHEA-S level was normalized to 3.4 μmol/L (125 µg/dL), and she showed an improved triglyceride level on her lipid panel. Ten months post left adrenalectomy, DST was reperformed and cortisol was appropriately suppressed (0.9 µg/dL).

## Discussion

ACTH-independent Cushing syndrome, resulting from unilateral adrenal tumors, is typically treated with unilateral adrenalectomy, which is often curative and considered the primary treatment approach. However, medical therapy is indicated if surgery is contraindicated, postponed, or ineffective (3). Mifepristone, approved by the Food and Drug Administration on February 7, 2012, serves as a once-daily oral medication aimed at managing hyperglycemia secondary to hypercortisolism in adults with endogenous Cushing syndrome and type 2 diabetes or glucose intolerance, particularly for those unsuitable for surgical intervention. Acting as a competitive glucocorticoid antagonist, mifepristone has demonstrated efficacy in restoring a normal hypothalamic-pituitary-adrenal axis and mitigating postoperative adrenal insufficiency (4).

To our knowledge, the discovery of lipomatous metaplasia in an adrenal cortical adenoma has been reported only once previously, in a 1996 case study by Feldberg et al (5). Their patient presented with a 9 cm left adrenal mass and an elevated DHEA-S level, with pathology revealing numerous foci of mature adipose tissue. Their final diagnosis was an adrenocortical tumor of uncertain malignant potential. Similarly, our patient also exhibited an elevated DHEA-S level; in the setting of low normal ACTH secretion, high DHEA-S may be secondary to cytochrome b5 overexpression stimulating 17, 20-lyase, raising the possibility that these tumors are both cortisol and androgen secreting. However, unlike the previous case, our patient displayed clinical and biochemical findings indicative of hypercortisolism.

In a study conducted by Selye and Stone (6) at the University of Montreal in 1950, lipomatous changes in the adrenal glands of rats were investigated following methyl testosterone injections. These researchers observed the development of notably large vacuoles containing sudanophilic lipid material in the adrenal cortex. They concluded that androgen compounds could induce the transformation of adrenal cortical cells into typical fat cells, with this effect being heightened when corticotropic anterior pituitary extracts were administered simultaneously. Selye and Stone's seminal work offers a potential mechanism to explain why our patient may have developed lipomatous metaplasia. Our patient had evidence of DHEA-S secretion, a compound with androgenic properties, as well as cortisol from her adrenal tumor. Subsequent treatment with mifepristone increased the release of ACTH from the anterior pituitary through competitive glucocorticoid antagonism, thus inhibiting the negative feedback loop and restoring the hypothalamic-pituitary-adrenal axis.

In their 1998 study on adrenal fat cell metaplasia (a postmortem series), Saeger and Reinhard (7) postulated that adipose metaplasia increases with age. These authors noted a correlation between adipose metaplasia incidence and the presence of arterial hypertension and severe coronary heart disease. The authors proposed that these foci of metaplasia likely develop due to ischemic lesions, possibly in combination with local endocrine hyperstimulation. They also theorized that the development might originate from macrophages that phagocytosed lipids from necrotic, lipid-rich adrenocortical cells. Therefore, factors other than androgen hormones could contribute to excessive fat storage in the adrenal cortex, leading to adipose metaplasia. Notably, our patient had hypertension, type 2 diabetes, morbid obesity, and dyslipidemia, all exacerbated by her hypercortisolism.

It is conceivable that the development of foci of lipomatous metaplasia in our patient's adrenal gland was induced by elevated androgen hormone secretion and/or hypercortisolism, which exacerbated her diabetes, hypertension, and dyslipidemia, thereby contributing to ischemic adrenal lesions. Furthermore, there may exist a correlation between her elevation of endogenous ACTH during mifepristone therapy in conjunction with these conditions, resulting in the cellular transformation leading to adipose metaplasia in the resected adrenal gland.

We presented a rare case of ACTH-independent endogenous Cushing syndrome arising from a left adrenal cortical adenoma with lipomatous metaplastic changes. Medical treatment with spironolactone and mifepristone served as a bridge until definitive surgical treatment was performed. Postsurgery, the patient displayed normalized DHEA-S and ACTH levels, as well as appropriate cortisol suppression with DST testing. Importantly, the patient also improved clinically. As metaplasia represents a preneoplastic process that can progress into dysplasia, a state with neoplastic potential, it is imperative to explore potential risk factors associated with this diagnosis.

## Learning Points

- Metaplasia represents a preneoplastic process that has the potential to progress into dysplasia.
- Adrenal lipomatous metaplasia may be triggered by androgen compounds and ACTH. It may also be associated with advanced age, systemic stress, vascular conditions, or metabolic diseases, likely due to ischemia.
- Surgical resection remains the primary treatment for functional adrenal tumors. However, in cases where medical therapy is required, clinicians should consider the risk factors associated with lipomatous metaplasia and understand the mechanism of action of the medication, as an increase in ACTH levels could potentially contribute to adrenal adipose metaplasia.
- Preoperative mifepristone treatment may shorten the posttreatment glucocorticoid replacement period in ACTH-independent Cushing syndrome, as evidenced by effective cortisol secretion neutralization without adrenal atrophy or postoperative glucocorticoid replacement requirement.

## Data Availability

Data sharing does not apply to this article as no datasets were generated or analyzed during the current study.
